# Melanoma risk factors in a Latin American population^☆^^☆☆^

**DOI:** 10.1016/j.abd.2019.11.009

**Published:** 2020-05-11

**Authors:** John Alexander Nova, Guillermo Sánchez-Vanegas, Mauricio Gamboa, Sebastian Ramiro Gil-Quiñones

**Affiliations:** Department of Skin Cancer Teaching and Research, Centro Dermatológico Federico Lleras Acosta, Bogotá, Colombia

Dear Editor,

Malignant melanoma (MM) is a skin tumor associated with a high mortality worldwide. The five-year survival rate is 95% if melanoma is detected early and only 5% for metastatic melanoma.^1^ In Colombia, the national registry reported an increased incidence of melanoma from four to six cases per 100,000 people in merely four years.^2^ This highlights the importance of identifying melanoma risk factors, especially in Latin-American countries were the distribution of histopathological subtypes of melanoma is divergent with the reports in other countries, where acral lentiginous melanoma (ALM) is the most frequent MM subtype.^3^ Although a possible association with trauma has been reported, this association has not been clearly demonstrated.^4^, ^5^

Considering the aforementioned, a case-control study was performed between 2010 and 2014 in the population seen at the Federico Lleras Acosta Dermatology Center, a dermatological referral hospital in Bogotá, Colombia. Data from patients histologically diagnosed with melanoma were collected. The controls were those patients admitted to the same hospital for non-melanoma dermatological disease or non-melanoma skin cancer. All controls underwent a questionnaire and physical examination verifying that they had neither melanoma nor lesions clinically suggestive of melanoma. The cases and controls were age-matched by approximately five years. Two controls were assigned to each case

Sociodemographic variables, history of working outdoors and outdoor sports participation throughout life, insecticide exposure, smoking, sunburn history, and a family history of skin cancer were studied. Individual phenotypic features including skin phototype, eye color, hair color, and signs of sun damage were also studied.

Associations through the chi-squared test, Student's *t*-test and Wilcoxon rank-sum were used for statistical analyses and a multivariate analysis using conditional logistic regression was performed, with statistically significant, clinically relevant, and potentially confounding variables included. Data were analyzed using the statistical software Stata.

This study included a total of 243 participants; 81 cases and 162 controls. The average subject age was 64 years. Analyzing the age by subtype, the patients with lentigo maligna averaged 67 years; the patients with acral lentiginous melanoma and nodular melanoma averaged 63 years; and those with superficial spreading melanoma averaged 58 years.

Of the total participants, 160 were women (66%) and 83 were men (34%). In the case group, the female percentage was 68% (55/81); in the control group, it was 65% (105/162). Table 1 shows the histologic classification of the tumors. The most common melanoma subtype was acral lentiginous melanoma (32%), followed by lentigo maligna (29%). The melanomas were located mostly on the cheeks 21/81 (26%), nails 14/81 (26%), nose 11/81 (13%), and the soles of the feet 9/81 (11%).

**Table 1:** Histologic melanoma subtypes in the studied cases.

Histologic subtype	Cases (*n* = 81)	
	*n*	%
*In situ melanomas*		
Lentigo maligna	24	29.62
Other *in situ* melanomas	9	11.11

*Invasive melanomas*		
Acral lentiginous melanoma	26	32.09
Nodular melanoma	10	12.34
Superficial spreading melanoma	4	4.93
Lentigo maligna melanoma	4	4.93
Non-categorized malignant melanoma	4	4.93

Nearly 73% (59/81) of the cases had completed secondary school, compared with 71% (115/162) of the controls, which was not a significant difference (*p* = 0.7 by chi-squared test).

Table 2 shows the results of the bivariate analysis which reveals that having worked outdoors during early adult life (15–30 years old) increased the risk of developing melanoma by 1.9 times. The most frequent occupations among cases and controls in this period were farming activities (54% *vs*. 67%), construction (5% *vs.* 4%), and outdoor sales (11% *vs.* 14%). No significant differences were found between both groups.

**Table 2:** Differences between variables related to sun exposure and phenotypic features for patients with melanoma and for controls.

Variable	Cases		Controls		*p*-Value (chi^2^)	OR	95% CI
	n	%	n	%			
Outdoor work between 15 and 30 years of age	37/81	46	49/162	30	0.01	1.93	1.07–3.48
Insecticide exposure	9/81	11	4/160	2.5	0.005	4.8	1.29–22.23
Outdoor sports after 30 years of age	15/81	18	58/162	36	0.00	0.4	0.19–0.80
More than 5 sunburns in life	18/81	22	4/162	2	0.00	11.28	3.49–47.13
More than 5 sunburns in childhood (<16 years old)	10/81	12	3/162	1.85	0.00	7.46	1.83–43.08
More than 5 sunburns between 15 and 30 years of age	18/81	22	13/162	8	0.001	3.27	1.41–7.70
More than 5 sunburns after 30 years of age	10/80	12	4/162	2	0.00	5.64	1.55–25.30
Personal history of skin cancer	3/80	4	0/162	0	0.03	.	.
Light eyes (green, blue, or hazel)	67/81	83	73/162	45	0.00	5.83	2.93–12.09
Light hair (blond, red, or brown)	47/81	58	38/162	23	0.00	4.51	2.44–8.31
Actinic conjunctivitis	25/79	32	19/162	12	0.00	3.48	1.68–7.24
Actinic comedones	2/79	2	19/162	12	0.01	0.19	0.02–0.84
Poikiloderma of Civatte	47/79	59	41/162	25	0.00	4.33	2.35–7.99
Numerous freckles	11/81	13	2/162	1	0.00	12.57	2.61–118.3
Numerous lentigines on face	10/81	12	6/162	4	0.01	3.66	1.14–12.68

The signs of chronic sun damage increased the risk of developing melanoma (Table 2). Phototypes 1 and 2 were more frequent in the case group – 51% (41/81) than in the control group – 42% (68/162); however, this difference was not significant.

History of insecticide exposure increased the risk of developing melanoma fourfold (OR = 4.8, 95% CI 1.29–22.23). Acral lentiginous melanoma was exhibited in 36% (4/11) of patients who reported exposure to pesticides. No significant differences between cases and controls were found in the other studied variables, such as smoking (35/81 *vs*. 72/162), sunscreen use (3/80 *vs.* 8/162), or the use of tanning beds (0).

Table 3 shows the risk factors identified after multivariate analysis, which included having blue or green eyes, actinic conjunctivitis, numerous freckles, and a history of ten or more sunburns throughout life.

**Table 3:** Risk factors for developing melanoma according to multivariate analysis.

Variable	OR	*p*	95% CI
Blue or green eyes	4.62	0.00	2.24–9.52
Actinic conjunctivitis	4.95	0.00	2.25–10.91
Numerous freckles	11.35	0.00	2.00–64.12
Ten or more sunburns throughout life	8.34	0.00	2.49–28.17
LR Chi: 77.58			
Pseudo *R*^2^: 0.25			

This study found that, although skin phototypes III and IV predominate in Colombia, phenotypic characteristics such as hair color, light eyes (green, hazel, or blue), as well as the number of freckles and the history of sunburn increase the risk of developing MM.

Consistent with previous studies, this population develops ALM as the predominant MM subtype. The association found between exposure to insecticides and risk of melanoma could strengthen the argument that this is one important element in the explanation of ALM pathophysiology.

## Financial support

This study was developed and funded entirely by the Federico Lleras Acosta Dermatology Center.

## Authors’ contributions

John Alexander Nova: Approval of the final version of the manuscript; conception and planning of the study; drafting and editing of the manuscript; collection, analysis, and interpretation of data; participation in study design; intellectual participation in the propaedeutic and/or therapeutic conduct of the studied cases; critical review of the literature; critical review of the manuscript.

Guillermo Sánchez Vanegas: Statistical analysis; approval of the final version of the manuscript; conception and planning of the study; drafting and editing of the manuscript; collection, analysis, and interpretation of data; participation in study design; intellectual participation in the propaedeutic and/or therapeutic conduct of the studied cases; critical review of the literature; critical review of the manuscript.

Mauricio Gamboa: Approval of the final version of the manuscript; conception and planning of the study; drafting and editing of the manuscript; collection, analysis, and interpretation of data; participation in study design; intellectual participation in the propaedeutic and/or therapeutic conduct of the studied cases; critical review of the literature; critical review of the manuscript.

Sebastian Ramiro Gil Quiñones: Approval of the final version of the manuscript; conception and planning of the study; drafting and editing of the manuscript; collection, analysis, and interpretation of data; participation in study design; intellectual participation in the propaedeutic and/or therapeutic conduct of the studied cases; critical review of the literature; critical review of the manuscript.

## Conflicts of interest

None declared.
